# Fluorescence Imaging Using Enzyme-Activatable Probes for Detecting Diabetic Kidney Disease and Glomerular Diseases

**DOI:** 10.3390/ijms23158150

**Published:** 2022-07-24

**Authors:** Kentaro Yamada, Tomoaki Takata, Takuji Iyama, Shintaro Hamada, Yukari Mae, Takaaki Sugihara, Hajime Isomoto

**Affiliations:** Division of Gastroenterology and Nephrology, Faculty of Medicine, Tottori University, Yonago 683-8504, Japan; k.damaya@gmail.com (K.Y.); raisei_ka_1227@yahoo.co.jp (T.I.); hamashin7650@gmail.com (S.H.); yuuchanfront@gmail.com (Y.M.); sugitaka@tottori-u.ac.jp (T.S.); isomoto@tottori-u.ac.jp (H.I.)

**Keywords:** diabetic kidney disease, diabetic nephropathy, nephrosclerosis, glomerulonephritis, enzyme-activatable probe, gamma-glutamyl transpeptidase, dipeptidyl-peptidase, fluorescence imaging, fluorescent probe

## Abstract

A clear identification of the etiology of glomerular disease is essential in patients with diabetes. Renal biopsy is the gold standard for assessing the underlying nephrotic pathology; however, it has the risk for potential complications. Here, we aimed to investigate the feasibility of urinary fluorescence imaging using an enzyme-activatable probe for differentiating diabetic kidney disease and the other glomerular diseases. Hydroxymethyl rhodamine green (HMRG)-based fluorescent probes targeting gamma-glutamyl transpeptidase (GGT) and dipeptidyl-peptidase (DPP) were used. Urinary fluorescence was compared between groups which were classified by their histopathological diagnoses (diabetic kidney disease, glomerulonephritis, and nephrosclerosis) as obtained by ultrasound-guided renal biopsy. Urinary fluorescence was significantly stronger in patients with diabetic kidney disease compared to those with glomerulonephritis/nephrosclerosis after DPP-HMRG, whereas it was stronger in patients with nephrosclerosis than in patients with glomerulonephritis after GGT-HMRG. Subgroup analyses of the fluorescence performed for patients with diabetes showed consistent results. Urinary fluorescence imaging using enzyme-activatable fluorescence probes thus represents a potential noninvasive assessment technique for kidney diseases in patients with diabetes.

## 1. Introduction

Diabetes mellitus is the leading cause of kidney disease, and approximately 40% of patients with diabetes develop chronic kidney disease (CKD) [1]. Diabetic kidney disease typically presents with albuminuria or proteinuria, which may reach the nephrotic range that induces a progressive decline in renal function [2]. It is diagnosed by two urinalysis-based tests: (i) estimated glomerular filtration rate (eGFR) and (ii) urine albumin or protein. However, abnormalities in the glomerular capillary filter due to glomerulonephritis, nephrosclerosis, or other pathologies can cause proteinuria. Since such glomerular diseases occur in patients with diabetes, a clear identification of the etiology is pivotal, especially in those with proteinuria. Renal biopsy is the gold standard for assessing kidney diseases; however, since it is invasive, clinicians need to carefully evaluate whether the benefits of undergoing renal biopsy outweigh the patient’s risk of potential complications. Therefore, an easy, convenient, and noninvasive method with more specificity is warranted for the diagnosis of kidney disease [3].

An enzyme-activatable probe was originally developed for the photodynamic diagnosis of cancer [4]. The detection of the enzymatic activity of aminopeptidases such as gamma-glutamyl-transpeptidase (GGT) and dipeptidyl-peptidase (DPP) is the basis for this diagnostic technique. Cells/tissues preserving aminopeptidase expression emit fluorescence upon incubation with the targeted activatable fluorescence probe. One of the most valuable applications of this probe is in detecting cancers preserving high enzymatic activities. Pancreatic cancer cells obtained by a fine-needle aspirated biopsy can be identified by GGT-activatable probes. Similarly, DPP-activatable probes can detect esophageal cancer in biopsy specimens [5]. Fluorescence imaging using this enzyme-activatable probe has a high sensitivity and shows a rapid fluorescence emission. Therefore, we hypothesized that urinary fluorescence imaging would be a novel noninvasive assessment technique for identifying kidney diseases in patients with diabetes. The aim of the study was to investigate whether urinalysis by fluorescence imaging using the enzyme-activatable fluorescent probe can be applied for patients with diabetes.

## 2. Results

**Figure 1:**
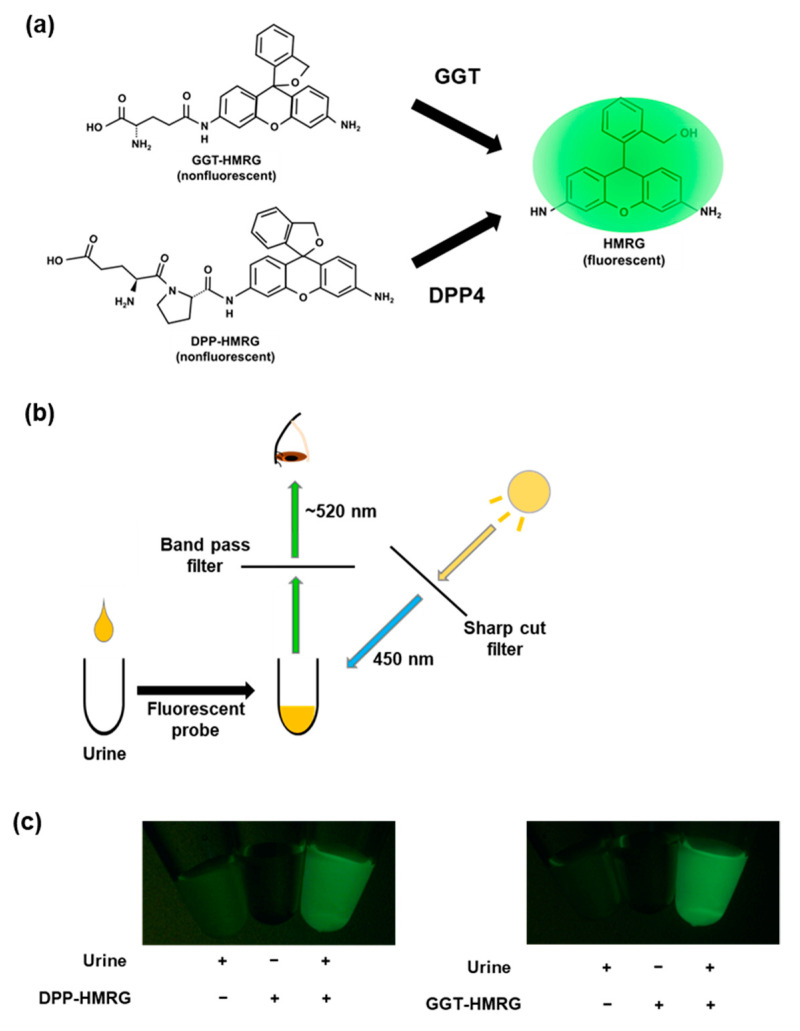
Urinary fluorescence analysis. (**a**,**b**) Scheme of fluorescence imaging using enzyme-activatable fluorescent probes. GGT-HMRG and DPP-HMRG are non-fluorescent but emit fluorescence upon activation by GGT or DPP. (**c**) Representative urinary fluorescence images after DPP-HMRG and GGT-HMRG. Although the urine showed a faint autofluorescence, remarkable fluorescence was observed after incubation with the fluorescent probes.

First, we investigated whether urinary fluorescence can be obtained after incubation with DPP-hydroxymethyl rhodamine green (HMRG) and GGT-HMRG. Urine from a healthy subject underwent fluorescence imaging using an optimized procedure. Although faint autofluorescence was detected in the urine, remarkable fluorescence could be detected after incubation with DPP-HMRG and GGT-HMRG (Figure 1).

We included 114 patients in this study; after excluding 12 patients: interstitial fibrosis (n = 7), drug-induced acute kidney injury (n = 2), and not diagnosed (n = 3), the urine samples of 102 patients were analyzed (Table 1). Fluorescence images after the application of two HMRG-based activatable probes, GGT-HMRG and DPP-HMRG, were obtained from all the examined samples. No significant correlations were observed in the fluorescence intensities with age, body mass index (BMI), eGFR, or urinary protein (Table 2). Diabetes mellitus and DPP-4 inhibitor medications did not affect the fluorescence intensities after GGT-HMRG or DPP-HMRG application.

**Figure 2:**
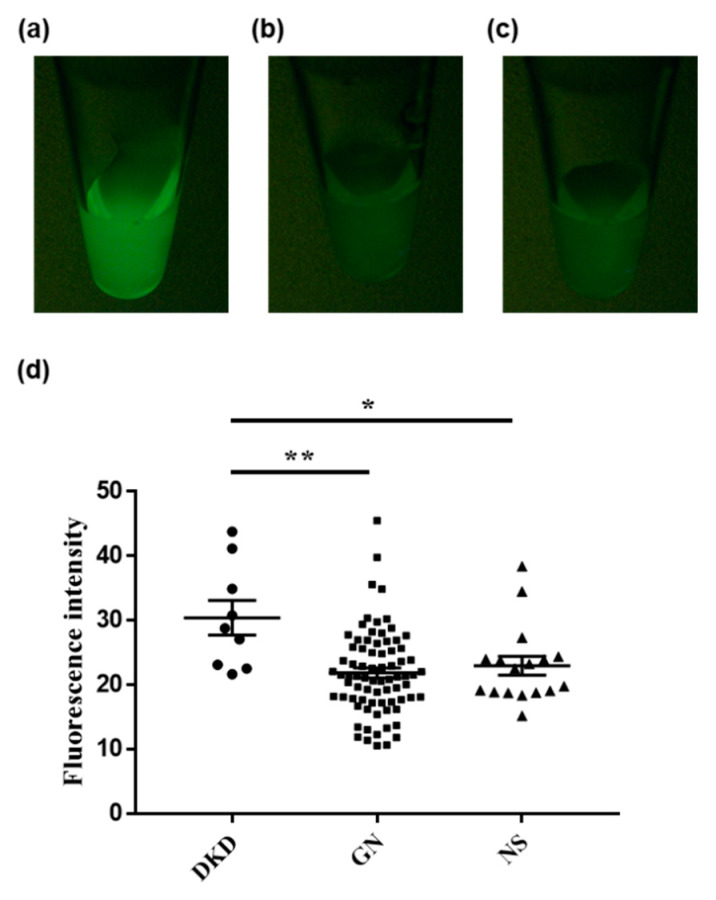
Fluorescence images of urine after incubation with DPP-HMRG. Representative fluorescence images of urine obtained from patients with diabetic kidney disease (**a**), glomerulonephritis (**b**), and nephrosclerosis (**c**) after incubation with DPP-HMRG. Urine from patients with diabetic kidney disease showed a significantly high fluorescence intensity compared to the other groups (**d**). * *p* < 0.05, ** *p* < 0.01.

**Figure 3:**
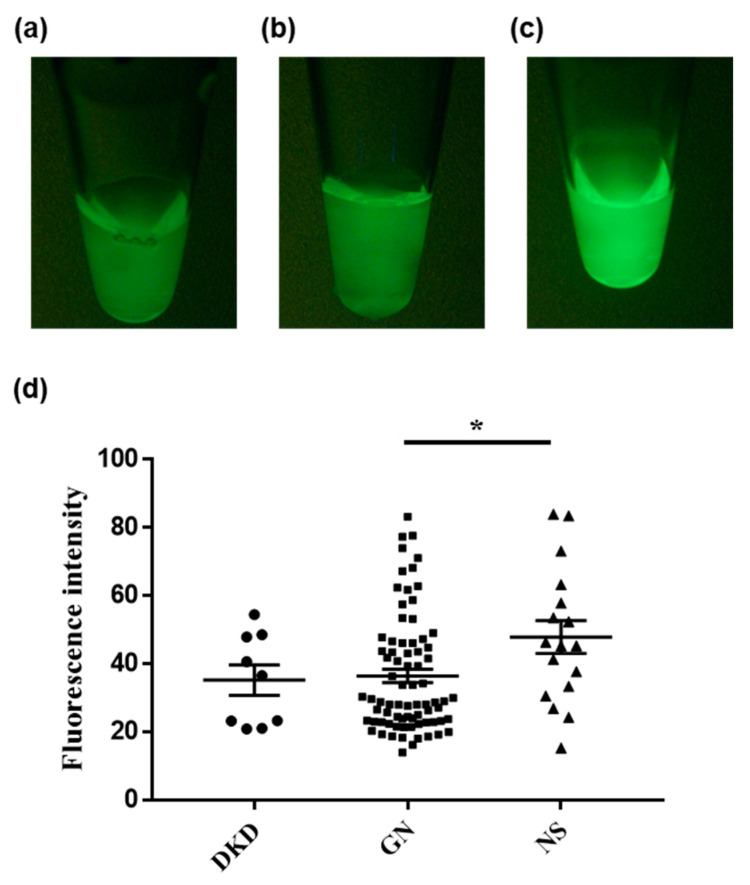
Fluorescence images of urine after incubation with GGT-HMRG. Representative fluorescence images of urine obtained from patients with diabetic kidney disease (**a**), glomerulonephritis (**b**), and nephrosclerosis (**c**) after incubation with GGT-HMRG. Urine from patients with nephrosclerosis showed a significantly high fluorescent intensity than those with glomerulonephritis (**d**). * *p* < 0.05.

**Figure 4:**
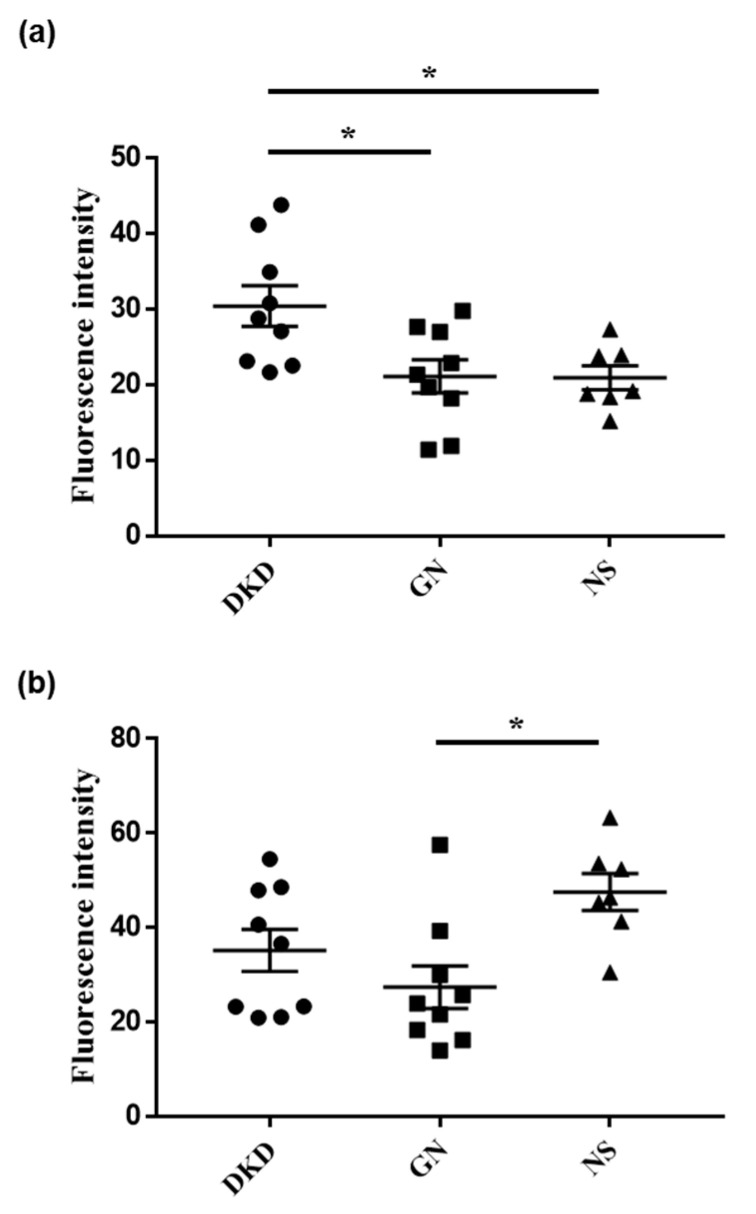
Fluorescent intensities of urine in patients with diabetes. Urinary fluorescent intensities after incubation with DPP-HMRG (**a**) and GGT-HMRG (**b**) in patient with diabetes. Diabetic kidney disease could be distinguished from the other groups by DPP-HMRG, whereas nephrosclerosis could be differentiated from glomerulonephritis by GGT-HMRG. * *p* < 0.05.

Next, we investigated whether the fluorescence intensities of the urine samples could indicate any underlying nephritic pathology. Urine from patients with diabetic kidney disease showed a significantly stronger fluorescence after DPP-HMRG application than urine from patients with nephrosclerosis/glomerulonephritis (Figure 2). Moreover, the fluorescence after GGT-HMRG incubation was significantly stronger in patients with nephrosclerosis than in those with glomerulonephritis (Figure 3). Finally, a subgroup analysis of the fluorescence performed for 25 patients with diabetes (Table 1) showed consistent results after DPP-HMRG and GGT-HMRG incubation (Figure 4).

## 3. Discussion

In this study, we demonstrated that an HMRG-based activatable fluorescence probe could be used for urine sample analysis. Urinary fluorescence after DPP-HMRG was stronger in patients with diabetic kidney disease than patients with nephrosclerosis/glomerulonephritis, whereas the fluorescence after GGT-HMRG was stronger in patients with nephrosclerosis than those with glomerulonephritis. We propose that urinary fluorescence imaging represents a novel diagnostic method for kidney disease.

Several urinary biomarkers such as *N*-acetyl-β-d-glucosaminidase, β2-microglobulin, and neutrophil gelatinase-associated lipocalin have been used as indicators of kidney injury. However, these are not specific for diabetic kidney disease. Diagnostic methods other than such biochemical analyses have been suggested as candidates for diagnosing diabetic kidney disease [6,7]; however, there is no established method for distinguishing diabetic kidney disease and other pathologies.

The feasibility of HMRG-based fluorescent probes for photodynamic diagnosis has mostly been investigated in detecting cancers. Reports using GGT-targeted fluorescent probes have focused on detecting cancers based on the abundant expression of GGT in cancer cells including hepatocellular carcinoma, ovarian cancer, and breast cancer [4,8,9]. This technique also has a broad application beyond cancers. We recently demonstrated that, based on the abundant expression of GGT in renal proximal tubules, renal biopsy samples could be rapidly evaluated by GGT-HMRG fluorescent probes [10]. In the present study, urine fluorescence could be detected after GGT-HMRG incubation, and the fluorescence was significantly higher in patients with nephrosclerosis than patients with glomerulonephritis. Urinary GGT level is a potential biomarker in acute kidney injury [11,12]. Furthermore, serum GGT levels are associated with endothelial dysfunction in patients with chronic kidney disease and are a predictor for end-stage renal disease [13,14]. A stronger GGT-HMRG fluorescence in nephrosclerosis may reflect renal atherosclerotic injury.

DPP-4 is localized to the glomerular podocytes and brush border of proximal tubular cells [15]. The tubular expression of DPP-4 increases in diet- or streptozotocin-induced diabetic rats [16]. Circulating soluble DPP-4 levels are high in patients with diabetes mellitus [17], and DPP4, which is bound to urinary microvesicles, can be useful for staging diabetic kidney disease [18]. However, sophisticated devices are required for measuring these enzyme activities.

Urinary fluorescence imaging, which uses enzyme-activatable fluorescence probes, can be performed even with a conventional digital camera [10]. Moreover, as the current nephrology practice guidelines emphasize bedside assessments of patients with diabetes and kidney diseases [19], urinary fluorescence imaging should be considered viable, since it is based on the high sensitivity and rapid fluorescence emissive power of enzyme-activatable fluorescence probes. Urinary fluorescence imaging is a unique and reasonable approach for bedside assessment in health care systems.

This study included only a small sample size; thus, this fluorescence imaging technique requires confirmation with larger studies. The applicability of urinary fluorescence imaging requires setting or investigating the quantitative thresholds along with the standard operating procedures and general inclusion/exclusion criteria in the future. Nevertheless, urinary fluorescence imaging is a potential noninvasive assessment technique for kidney diseases that is applicable for patients with diabetes.

## 4. Materials and Methods

### 4.1. Study Population

This study included patients who underwent ultrasound-guided renal biopsy at Tottori University Hospital. The renal biopsies were performed for patients with suspected glomerular diseases [20,21]. Urine was collected before the renal biopsy and stored at −80 °C until analysis. Renal biopsy tissues were fixed in 10% formalin and embedded in paraffin. Periodic Acid–Schiff staining of the 4-µm thick sections was performed by experienced nephrologists who were blinded to the fluorescence image analysis. The BMI, serum creatinine, and urinary protein levels of the patients were measured. We determined that a total number of 84 participants would provide the study with 90% power (*p* = 0.05; effect size: 0.40). The target number of participants was calculated using software (G*Power version 3.1.9.6, Buchner A, Düsseldorf, Germany).

### 4.2. Activatable Fluorescent Probe

Two fluorescent probes containing HMRG were purchased (GC811-EP-HMRG and GC801-ProteoGREEN-gGlu, GORYO Chemical, Sapporo, Japan) to target DPP and GGT. Both EP-HMRG and GGT-HMRG have a high specificity for DPP and GGT [4,5]. Each probe has been well characterized by nuclear magnetic resonance spectroscopy or high-performance liquid chromatography and documented previously [4,5]. The DPP-HMRG and GGT-HMRG fluorescence probes were prepared as per the manufacturer’s instructions and stored at −30 °C until usage. The stock solutions for each fluorescent probe were thawed on ice and diluted in phosphate-buffered saline and incubated with the urinary samples.

### 4.3. Fluorescence Imaging

We investigated the optimal conditions for fluorescence imaging. Freeze-thaw cycles of the urine samples and the temperature of incubation did not affect the fluorescence intensities. The fluorescence depended on the concentrations and pHs of the samples. Therefore, urine was adjusted according to the creatinine concentration with phosphate-buffered saline. Stored urine was thawed on ice and diluted in phosphate-buffered saline to a creatinine concentration of 1 μg/μL to equalize urine concentration and pH. In this way, the influence of urine concentration and pH could be minimized sufficiently. The fluorescence solutions were added to each urine sample at a final concentration of 5 µM and incubated for a minute. The fluorescence images were obtained by following an optimized procedure. In brief, the mixture of the sample and the fluorescence probe was exposed to an excitation wavelength of 450 nm, and then the fluorescence images were obtained by a stereomicroscope (catalog number: BS-3048BT, BioTools, Takasaki, Japan) equipped with an optical interference filter (~520 nm). Target regions of interest were set for the fluorescent images with digitally obtained images; thereafter, the fluorescence intensities were measured using the ImageJ software (U.S. National Institutes of Health, Bethesda, MD, USA).

### 4.4. Statistical Analysis

Continuous variables were expressed as the mean ± standard deviation or median with interquartile range according to the distribution. The Kolmogorov–Smirnov test was used to assess the normal distribution. Correlations between fluorescence intensities and clinical parameters were analyzed by Pearson’s correlation coefficient. Differences between two groups were analyzed using Welch’s t test. Differences between more than three groups were analyzed by one-way analysis of variance with a post-hoc Tukey test. A *p*-value of less than 0.05 was considered statistically significant. GraphPad Prism (7.0. for Windows, GraphPad Software, San Diego, CA, USA) was used for the analysis.

## Figures and Tables

**Table 1:** Patients’ characteristics.

	All	DM (+)
Number	102	25
Sex (male/female)	59/43	19/6
Age, years	55.3 ± 20.7	62.5 ± 13.0
Body mass index, kg/m^2^	23.3 ± 4.2	25.8 ± 5.1
Creatinine, mg/dL	0.93 (0.38–8.71)	1.10 (0.45–8.71)
estimated GFR, mL/min/1.73 m^2^	61.6 ± 32.3	47.9 ± 26.8
Urinary protein, g/day	0.93 (0.05–16.47)	1.79 (0.20–10.52)
Histological diagnosis		
Glomerulonephritis, n	76	9
Nephrosclerosis, n	17	9
Diabetic kidney disease, n	9	7

DM, diabetes mellitus; GFR, glomerular filtration rate.

**Table 2:** Correlations between fluorescent intensities and clinical parameters.

	GGT-HMRG		DPP-HMRG	
	r	*p*-Value	r	*p*-Value
Age, years	0.041	0.69	0.133	0.18
Body mass index, kg/m^2^	0.121	0.26	0.010	0.93
eGFR, mL/min/1.73 m^2^	0.030	0.77	−0.086	0.39
Urinary protein, g/day	0.039	0.70	0.180	0.072

GGT, gamma-glutamyl-transpeptidase; DPP, dipeptidyl-peptidase; HMRG, hydroxymethyl rhodamine green; eGFR, estimated glomerular filtration rate.

## Data Availability

This study’s datasets are available upon reasonable request.
